# Mr. Hill on Ptyalism

**Published:** 1812-04

**Authors:** George Nesse Hill

**Affiliations:** Medical Surgeon. Chester


*To the Editors of the Medical and Physical Journal.*

GENTLEMEN,

INORDINATE action of the salivary glands, has become a far more common occurrence in the low form of insanity, than it appears to have been previous to the very free use of mercurial remedies, designed to remove supposed visceral obstructions, particularly of the liver; in this most formidable of diseases, Ptyalism is a troublesome disgusting symptom, and very difficult to remove, even after the cause which operated to its production seems to be nearly subdued; and, where no predisposition to madness exists, and morbid action has once taken full possession of that part of the system destined to furnish the saliva, it does not appear from the annals of practice to be so much under the control of common remedial means as the other profluvia.

In mild typhus, and some diseases which evince a more particular connexion with the nervous system than others,

 salivation salivation sometimes appears to be spontaneous and critical; it is occasionally yet rarely so in Melancholia, more frequently marking the decline of Mania; every case classible under the former designation, may justly be considered a case of "diminished nervous influence." (Vide Mr. Davis' Account, Med. and Phys. Journal, No. 154, p. 452.) But this gentleman says nothing of the state of the catamenial discharge in his patient, though from the account of her age (45) there seems cause to apprehend that the temporary salivation formed one of those multifarious symptoms, which so frequently exist in such subjects, to confound the wisdom of the most acute theorist, and puzzle the steadiest practitioner. I mention this circumstance, because the uterine evacuation is, at this period of life, so often connected with other discharges, as to seem to ebb and flow alternately with them; that is, to be redundant or scanty, as others are increased or diminished; and this state will occasionally continue to distress the patient for a long period, even after the final menstrual cessation has taken place.

In dressing a large scrophulous ulcer on the foot, a few mornings ago, for a female precisely thus situated, (æt. 48,) who has latterly labored under all the inconvenient distressing circumstances usually attendant upon redundant departing menses, but who had missed the two last returns, a sudden unusual tumefaction of the edges of the ulcer, with an abundant discharge of thin sordid fluid from its surface, far beyond the accustomed quantity, and before the succeeding dressing the coverings of the part were drenched in sanguinary discharge; this sufferer has also great inconvenience from occasional ptyalism, when any cause operated to diminish the excessive uterine evacuation.

Mr. D. wishes to obtain some information "respecting the connexion subsisting between the nervous system in its morbidly deranged state, and the salivary glands of the mouth and throat:" for, says he, "I have since observed the symptoms alluded to in a slighter degree occurring in patients of the same cast." General unequal excitement, and morbidly increased action in some important organ, being with wonderful celerity removed to another with a correspondent degree of diminished action in some no less important part, is a fact furnished by daily experience: witness the feet of marble coldness, the burning head and throbbing temples of the maniac; the distended spleen, with indescribable sensation and shrinking torpid surface of the melancholic: such are the common phenomena attendant on what are called nervous diseases, at the head of which madness stands with appalling pre-eminence. Particularly is this the case with respect respect to females; and of this sex those who are most prone to irregularities in the pelvic viscera, are most liable to those irregularities in the nervous system which are often so difficult to account for, and not less so to remove, or even to mitigate; hence all the secretions suffer in their turn.

A few months past, a young woman experienced very dreadful unaccountable Ischuria, with long-existing Amenorrhœa. During the constant use of the catheter, and remedies intended to relieve the uterine mischief, a rending pain of the head came on, with frequent epistaxis; but, although the blood discharged by the nose was considerable, the pain was but a little relieved, until a sudden and excessive ptyalism commenced, which appeared to remove it entirely. This belief was confirmed by the salivary evacuation abruptly stopping, and the pain returning. Blistering the pubes, opium, saline aperients, tinctura lyttæ, with emollient enemas and fomentation, succeeded in assisting to restore the urinary function; the catamenia returned, and the ptyalism receded with the other symptoms. But there are few of the numerous and distressing situations to which the human frame is exposed, that afford such striking changes and inexplicable phenomena as insanity, and, amongst a multitude of others, profuse salivation. Sometimes a patient, suffering under the low form of this complaint, has a quantity of fluid evacuated from the appropriate glands that would be incredibly astonishing, could we forget the well-known experiment of the late Dr. Monro.

A gentleman came under my care laboring under severe melancholia; he had been previously ill four months, during which period an almost uninterrupted ptyalism had existed. Upon devoting much time and strict attention to his situation, I found that the spitting was accompanied with a peculiar automatic motion of the left side of the mouth, with certain curious vibratory motions of the arm and leg of the same side, and that these actions always accompanied each other, that of the lips resembled a person drawing them sideways and backwards with a noise and jerk to extract food sticking between the molar teeth of that side; this movement was instantly succeeded by another, corresponding exactly in noise and manner with that of a smoker when puffing out the vapor from the middle of the lips; the affected upper limb was drawn upwards and downwards, the foot twitching outwards at the same time. After twelve months arduous trial to remove the cause of all the evils this unhappy man endured, but small progress appeared to have been made: as almost a last attempt, a large seton was introduced high up in the neck; and, as soon as it discharged freely, freely, the patient was placed in an entirely new situation, commanding delightful scenery. He took graduated doses of opium and hyoscyamus every three hours, with the intermission of only seven hours out of each twenty-four; this plan was followed for many weeks, by which means, and saline aperients, the melancholia receded, the ptyalism with its accompaniments disappeared, and, excepting an occasional slight recurrence of the peculiar labial motion, he perfectly recovered.

Serious and perplexing results have arisen from ill-applied powerful astringents, calculated, or at least intended, to suppress ptyalism, where due attention has not been devoted to the investigation of the cause or causes of the general mischief, of which this was merely a troublesome symptom; but on this subject, and on others connected with it, an opportunity will soon be afforded me of giving publicity to some (as I conceive) important observations, in an attempt to point out the path to greater success in the cure of insanity, than has hitherto obtained. I am, Gentlemen,

Your obliged Servant,

GEORGE NESSE HILL,

*Chester, Jan.* 1, 1812. Medical Surgeon.

